# Serial disparity in the carnivoran backbone unveils a complex adaptive role in metameric evolution

**DOI:** 10.1038/s42003-021-02346-0

**Published:** 2021-07-15

**Authors:** Borja Figueirido, Alberto Martín-Serra, Alejandro Pérez-Ramos, David Velasco, Francisco J. Pastor, Roger J. Benson

**Affiliations:** 1 Departamento de Ecología y Geología, Facultad de Ciencias, Universidad de Málaga, Málaga, Spain; 2 Departamento de Anatomía y Radiología, Museo de Anatomía, Universidad de Valladolid, Valladolid, Spain; 3 Department of Earth Sciences, University of Oxford, Oxford, UK

**Keywords:** Zoology, Evolution

## Abstract

Organisms comprise multiple interacting parts, but few quantitative studies have analysed multi-element systems, limiting understanding of phenotypic evolution. We investigate how disparity of vertebral morphology varies along the axial column of mammalian carnivores — a chain of 27 subunits — and the extent to which morphological variation have been structured by evolutionary constraints and locomotory adaptation. We find that lumbars and posterior thoracics exhibit high individual disparity but low serial differentiation. They are pervasively recruited into locomotory functions and exhibit relaxed evolutionary constraint. More anterior vertebrae also show signals of locomotory adaptation, but nevertheless have low individual disparity and constrained patterns of evolution, characterised by low-dimensional shape changes. Our findings demonstrate the importance of the thoracolumbar region as an innovation enabling evolutionary versatility of mammalian locomotion. Moreover, they underscore the complexity of phenotypic macroevolution of multi-element systems and that the strength of ecomorphological signal does not have a predictable influence on macroevolutionary outcomes.

## Abstract (web summary)

Figueirido et al. use a 3D geometric morphometric approach to study functional among-species disparity in the vertebral column of Carnivora, as well as assessing the effect of different sampling methods on homology. Disparity is generally higher in more caudal regions, compared to more cranial regions, but recruitment for locomotor function is pervasive throughout the whole studied column.

## Introduction

The regionalisation of the vertebral column into semi-autonomous modules has been acquired to different degrees in different groups of tetrapods^1^, giving rise to a spectacular phenotypic disparity ranging from aquatic dolphins to flying pterosaurs^2,3^ during the last ~350 million years. Indeed, the evolution of the tetrapod vertebral column is a classic example of evolutionary “trade-off” between flexibility in count—i.e. meristic variation—and regionalisation—i.e. homeotic variation^4–9^: the price for being regionalised is a lack of flexibility in count, but it allows selective responses in one module without generating non-adaptive morphologies in another^10^.

Recent quantitative studies have proposed that the ancestral amniote condition, despite being meristically variable, presents a subtly expressed regionalisation—or cryptic—due to the global-patterning *Hox* genes^11^. In contrast, although mammals show relatively invariant vertebral counts^1,6–10^, both their number of regions and the amount of morphological disparity between regions (heterogeneity) has increased along their evolutionary history^12,13^. Indeed, therian mammals show highly regionalised vertebral columns with a cervical region and with a trunk that is subdivided into rib-bearing thoracic and rib-less lumbar regions^13^. The origin of a specialised lumbar region in mammals is an evolutionary innovation related to novel *Hox* gene expression patterns, mostly related to *Hox10*^14^, and resulted in an increase in evolvability and ecological plasticity^13^.

The evolution of the vertebral column has been a research focus of many evolutionary biologists because the formative roles of somitogenesis, somatic growth and *Hox* gene expression are coupled with easily observable adult phenotypes^1^. However, the vertebral column poses greater analytical difficulties than the study of any single bone^15–17^ or of those structures composed of rigidly articulated bones such as the skull^18–20^, pelvis^21^ or the sacrum^22^ because there is not a clear criterion about the homology among subunits across taxa with different counts. Indeed, this is due to variation in the rate of somitogenesis or to variation in the expression of *Hox* genes along the antero-posterior axis of the embryo^1^. Changes in the rate of somitogenesis—i.e. velocity changes in a molecular oscillator (segmentation clock) that triggers the budding of a new somite from the presomitic mesoderm—results in meristic variation, and changes in *Hox* gene expression results in shifts of boundaries among regions, changing the number of somites that belong to each particular region^1^. These variations lead to difficulties in studies performed at interspecific level, as only homologous subunits (i.e. vertebrae) should be, a priori, compared across species.

Despite this, different among-species studies have investigated the evolution of metameric structures, and more particularly, the vertebral column^23–33^. For example, Randau and Goswami^34^ analysed the presacral column of a single family (Felidae) with fixed counts (7 C + 13 T + 7 L), and hence ensuring the homology of the subunits compared across taxa. On the other hand, Jones et al.^13^ analysed a phylogenetically wider dataset (52 mammalian species) with variation in count across taxa, but they solely used five thoracolumbar vertebrae for which homology across species was clear. These pioneering studies have been performed at a small phylogenetic scale such as within a single family^31,32,34,35^ or at a wider phylogenetic scale but restricted to a pre-specified subset of vertebrae^13^. However, at present, there is no formal quantification of the importance of morphological variation within regions (above the family level) due to problems related to homology^13,32,34^. This leaves important gaps in our knowledge on the evolution of multi-element phenotypes, i.e. the extent to which disparity of vertebral form is structured by ecomorphological adaptation, serial variation and evolutionary constraints, and the implications of how these aspects interplay for understanding evolutionary relationships between form and ecology in multi-component systems such as the vertebral column.

**Fig. 1:**
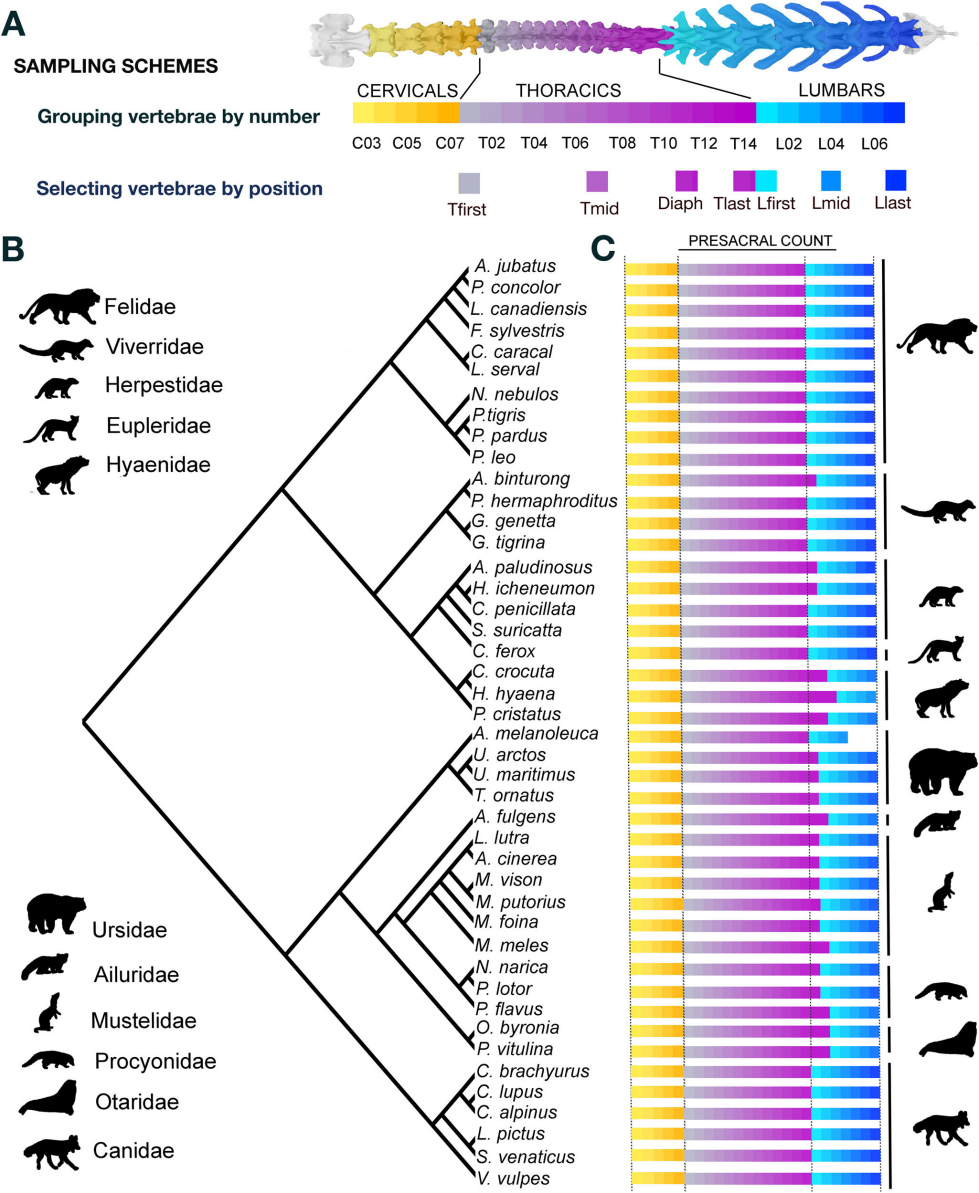
Phylogenetic relationships, sampling scheme and count variations across species used in this study. **A** Both sampling schemes performed in this study. Note that under the scheme of selecting vertebrae by position, the cervicals are also considered, but as all the taxa have a fixed cervical count at seven (as almost all mammals), they do not differ in both approaches. **B** Phylogenetic tree topology used in this paper. The tree topology and branch lengths in million years before present were taken from ref. ^44^. **C** The presacral count for the species analysed in this study is also given. In those species with homeotic variation (i.e. 13 T/7 L or 14 T/6 L) among different specimens (i.e. *Mustela putorious*, *Suricata suricatta*), the more conservative vertebral formula (vertebral count of its close relatives) is represented. Silhouettes not to scale. Sourced from Phylopic.org, all under public domain, except the silhouette for Procyonidae (*Nasua nasua*; http://phylopic.org/name/a94280d0-0db3-4a55-bcba-0cec7fdf6d51) by Rebecca Groom, and for Canidae (*Cuon alpinus*; http://phylopic.org/image/0bdb6532-0fa9-4ae7-992c-5b04a58d04d2/) by AnAgnosticGod (vectorized by T. Michael Keesey), both under Creative Commons Attribution-ShareAlike 3.0 Unported licence. The license terms can be found at: https://creativecommons.org/licenses/by-sa/3.0/.

Here, we analyse all the vertebrae of the presacral column by number, as well as a subset of selected vertebrae by position (Fig. 1A) in representatives of most of the families within the order Carnivora (Fig. 1B) to quantify both local-scale (within regions) and broad-scale (among regions) morphological variations in multi-element and functionally versatile phenotypes. Our analyses are based on a comprehensive dataset of 1097 presacral vertebrae (all presacrals except the atlas and axis) of 44 mammalian carnivores (Fig. 1B), whose members exhibit homeotic variation in the thoracolumbar portion of the presacral column—i.e. different numbers of thoracics and lumbars but keeping constant the thoracolumbar count at 20 (Fig. 1C)^8,9,36^. This approach to calculate among-species disparities “grouping vertebrae by number” offers a new avenue for analysing within-region variations that cannot be achieved without consideration of the whole column (Fig. 1A). However, the homology of some comparisons among subunits is less clear. To avoid biases in disparity calculations due to non-homologous comparisons, we also analyse a selected subset of individual vertebrae “by position” (Fig. 1A) for which regional homologies seem to be more clearly established^13^.

We specifically investigate: (i) among-species disparities for each vertebra of the presacral column “grouping vertebrae by number” and “selecting vertebrae by position”; (ii) the extent of vertebral morphospace partitioning among subclades through time, which provides evidence of the strength of constraints on morphological evolution, using both sampling schemes; (iii) the association between vertebral morphologies and the ecological habits of the sampled species “grouping vertebrae by number” and “selecting vertebrae by position”; and (iv) the comparison between the results obtained from the whole-column analyses grouping by number and from the subset of individual vertebrae selected by position to provide a firm basis for analysing serially homologous structures across different taxa with meristic and/or homeotic variations.

Our analyses of local-scale morphological variation between adjacent subunits of the same region demonstrate that the strength of the ecomorphological signal does not have a predictable influence on either disparity or constraint. Moreover, cervical and anterior thoracic regions are more adaptable than suggested by previous analyses that used sparse anatomical samples. Ecological adaptations of cervical and anterior thoracic vertebrae may be masked by a highly constrained low phenotypic variability, resulting in a one-to-many mapping pattern of evolution—i.e. the same morphological solution meet several adaptive challenges. The similarity between the results obtained from both sampling schemes (i.e. grouping vertebrae by number and selecting vertebrae by position) suggests a robust pattern across seriation in the carnivoran backbone. Only for some pairing comparisons among subunits the results of both approaches disagree, but this is mainly due to sampling differences between both schemes rather than to an artificial inflation of disparity values for wrong assumptions of homology. Current methods to quantify serially homologous structures and their assumptions regarding homology are discussed, and we provide a basis to analyse serial disparities in metameric structures with variation in count.

## Results

### Principal component analyses

**Fig. 2:**
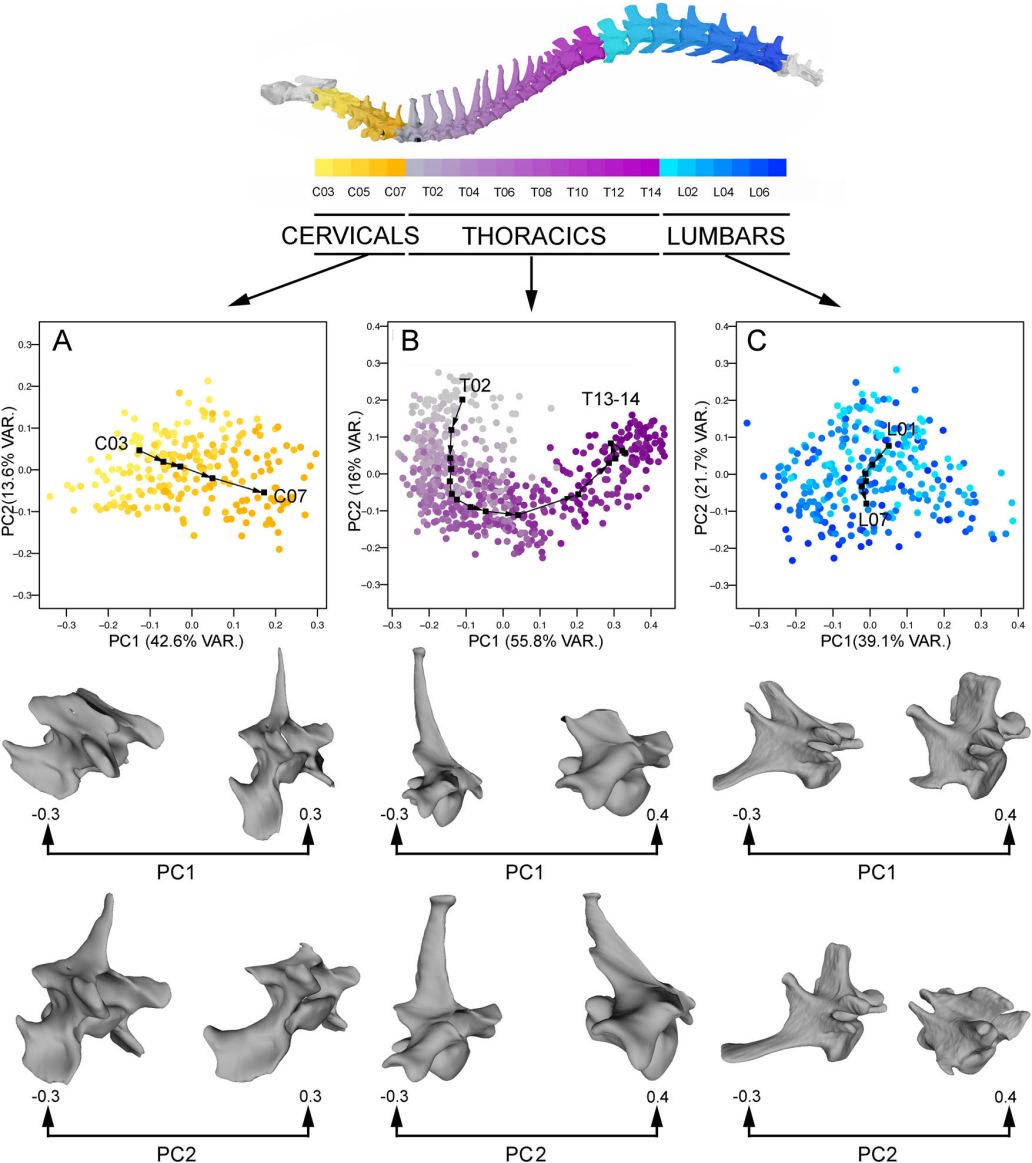
Results obtained from Principal Components analyses. Morphospace depicted by the bivariate graph of PC1 vs PC2 for the cervical (**A**), thoracic (**B**) and lumbar (**C**) regions. The 3D models showing the morphological changes associated with PC1 and PC2 at the extremes of each axis are shown for the three analyses. Black arrows represent the mean shape for each vertebra.

Principal component analysis (PCA) from landmark coordinates demonstrates variation in the degree of morphological individualisation among cervical, thoracic and lumbar vertebrae (Fig. 2A). Cervical and thoracic vertebrae have interspecific morphospaces with a relatively smaller degree of overlap between distributions of each element, whereas lumbars, and some posterior thoracics, show substantial overlap with each other (Fig. 2A).

The seventh cervical vertebra (C07) shows the greatest separation from others, while C04 and C05 overlap almost completely. Morphological traits distinguish among cervical morphologies (predominantly along PC1 and PC2) and mainly reflect variation in the length of the vertebral body and spinous process (PC1) and in the length and width of the vertebral body and of the spinous process (PC2) (Fig. 2A).

Accordingly, more anterior cervical vertebrae (e.g. C03) have negative PC1 scores—indicating longer bodies and shorter spinous processes—and positive PC2 scores—indicating relatively short, wide bodies and short spinous processes. More posterior vertebrae (e.g. C07) have positive PC1 scores—indicating shorter bodies and longer spinous processes—and negative PC2 scores—indicating relatively long, narrow bodies and long spinous processes (Fig. 2A).

The morphospace depicted by PC1 and PC2 for thoracic vertebrae is shown in Fig. 2B. PC1 separates mainly the first and the last thoracic vertebrae and PC2 separates the central vertebrae from the extreme ones, showing a clear “horse-shoe” effect. PC1 is associated with a morphological change in the relative size and length of the spinous process compared with the body (Fig. 2B). Therefore, more cranial vertebrae (approximately from T01 to T08) have negative scores, with long spinous processes and relatively small bodies (Fig. 2B) and the last vertebrae (approximately from T11 to T16) have positive scores, with short spinous processes and a relatively large body (Fig. 2B). PC2 is associated with the length of the vertebral body and the curvature of the spinous process. Central vertebrae (approximately from T07 to T10) have long bodies and caudally curved spinous processes (Fig. 2B) and extreme vertebrae (approximately from T01 to T03 and from T13 to T16) have short vertebral bodies and straight spinous processes (Fig. 2B).

The morphospace depicted from PC1 and PC2 for the lumbar region shows that the different vertebral positions overlap (Fig. 2C). The morphological changes associated with negative scores on PC1 correspond to long vertebral bodies, cranially oriented spinous processes and long transverse processes with a ventral and cranial orientation (Fig. 2C). Positive scores of this PC indicate short vertebral bodies, vertical and straight spinous processes and relatively short transverse processes with a lateral and horizontal orientation (Fig. 2C). For PC2, negative scores indicate short vertebral bodies, vertical spinous processes and long transverse processes with a cranial orientation (Fig. 2C). Positive scores indicate long vertebral bodies, cranially oriented spinous processes and very short transverse processes (Fig. 2C).

Moreover, this pattern is confirmed with the Procrustes analysis of variances (ANOVAs) grouping vertebrae by position and performed for each region. The morphology of the three regions is significatively influenced by position. However, the *Z*-score indicates that the magnitude of morphological variation according to vertebral position varies among regions, being lower for the lumbars than for the cervicals and thoracics (Table 1).

**Table 1:** Results of the three Procrustes ANOVAs for the three regions and grouping by vertebrae.

	D.f.	SS	MS	Rsq	*F*	*Z*-score	Pr(>*F*)
Cervical							
Vertebrae	4	2.991	0.748	0.322	25.479	**10.214**	0.001
Residuals	215	6.310	0.029	0.678			
Total	219	9.301					
Thoracic							
Vertebrae	15	24.261	1.61743	0.572	52.285	**16.113**	0.001
Residuals	586	18.128	0.03094	0.428			
Total	601	42.389					
Lumbar							
Vertebrae	6	1.9853	0.331	0.143	7.434	**7.888**	0.001
Residuals	268	11.929	0.045	0.857			
Total	274	13.914					

Values of *Z*-scores are in bold.

### Interspecific disparity

**Fig. 3:**
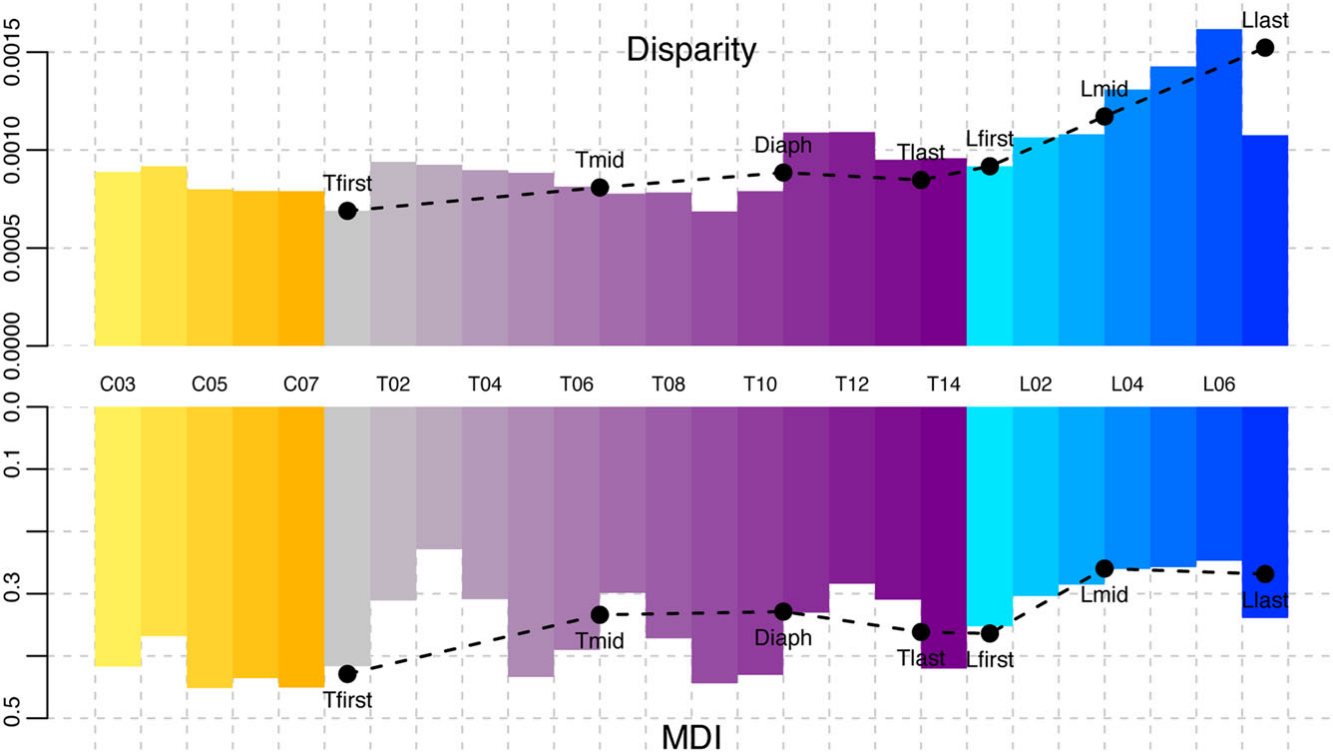
Disparity analyses. The upper histogram shows the disparity values for each vertebra among-species grouping vertebrae by number. The lower histogram shows morphological disparity index (MDI) for each vertebra grouping vertebrae by number. Points in both histograms indicate disparity and MDI values obtained using a selected set of vertebrae by position: Tfirst, first thoracic; Tmid, intermediate thoracic; Diaph, diaphragmatic vertebra; Tlast, last thoracic; Lfirst, first lumbar; Lmid, intermediate lumbar; Llast, last lumbar. The values for the cervicals are identical for both sampling schemes, as they do not differ in count across species. Along the *x*-axis, they are located close to their most frequent positions.

(i) *Grouping vertebrae by number*: for the analysis that includes all presacral vertebrae, morphological disparity (standardised by the number of digitised landmarks) varies substantially along the column with high disparity of lumbar morphologies, and relative low disparity of cervicals and anterior thoracics (Fig. 3 and Supplementary Table 1). Slight decreasing trends are evident from C04 to C07, and from T02 to T09. A remarkable increase in disparity is evident from T10 to T12 followed by a decrease in T13 to T14. Strikingly, disparity values among lumbar vertebrae exhibit a continuous increase from L01 to L06 to finally decrease in the last lumbar (L07). Because of this, L05–L07 exhibit the largest interspecific disparities of any vertebral positions (Fig. 3 and Supplementary Table 1).

(ii) *Selecting vertebrae by position*: using the subsampling approach—i.e. selecting some key vertebrae according to their position or anatomical relevance such as the first, intermediate and last thoracics (Tfirst, Tmid, Tlast), the diaphragmatic vertebra (Tdiaph) and the first, intermediate and last lumbars (Lfirst, Lmid, Llast) (see “Methods”)—the pattern drawn by the values of interspecific disparity across the axial system is confirmed (Fig. 3 and Supplementary Table 1). Therefore, a relatively low disparity in the Tfirst, Tmid and Tlast with a slight increase at the Tdiaph, and a continuous increase along the Lfirst, Lmid and Llast is noticed. The only exception of dissimilarity between the results obtained from both types of analyses is the disparity values for the Llast/L07, because while Llast is the most disparate vertebrae, the value of disparity for L07 is substantially lower than in other lumbars (Fig. 3 and Supplementary Table 1).

### Disparity through time analyses

Disparity through time analysis records the disparities of subclades of a given age relative to the whole-clade morphospace, and summarises the extent to which these patterns differ from expectations under a null model of evolution via the morphological disparity index (MDI). Generally, high MDI values are evident for carnivoran vertebral evolution, indicating that subclades occupy a large proportion of the whole-clade space, symptomatic of constrained evolution.

**Fig. 4:**
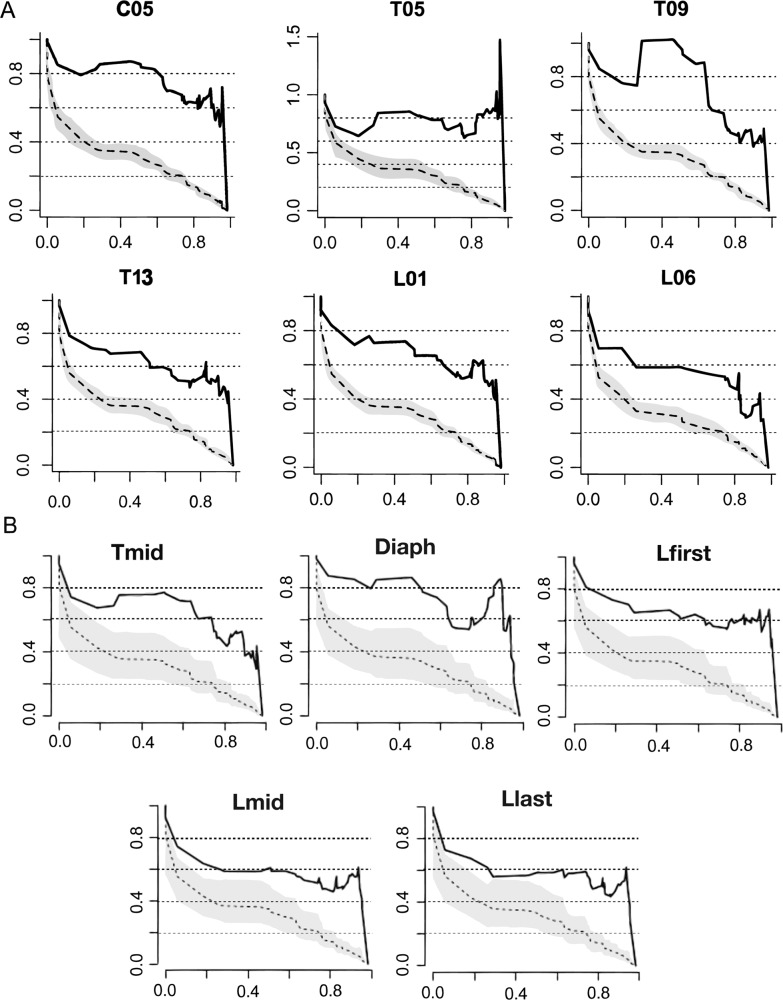
Disparity through time (DTT) plots for some selected vertebral shape using the residuals from allometric regression. **A** Results obtained grouping by vertebra for C05,T05, T09,T14, L01 and L06. **B** Results obtained selecting vertebrae by position for Tmid, Diaph, Lmid and Llast. Note that the results for Tfirst and Lfirst are the same than for T01 and L01 for the analysis grouping by vertebrae. The *Y*-axis is the average subclade disparity expressed as a proportion of total clade for subclades originating at different times (*X*-axis). Relative time values are used with 0.0 representing the root age and 1.0 representing the present. The solid line indicates the mean DTT from the empirical dataset, and the dashed line the simulated datasets. Dashed lines indicate the 95% DTT range (grey) and central tendency (black) for the simulated multivariate data under Brownian motion. For DTT plots of all the vertebrae see Supplementary Figure 1.

(i) *Grouping vertebrae by number*: All vertebrae exhibit high MDI values (Fig. 3 and Supplementary Table 1) ranging from 0.24 (in T03) to 0.47 (in C07), and disparity through time curves consistently show a high proportional subclade disparity through most of the evolutionary history of Carnivora (Fig. 4A and Supplementary Figure 1). The cervicals (C03–C07) and some thoracics (T01, T05–T06, T08–T10 and T14) exhibit comparatively high MDI. The lumbars and some other thoracics (especially T02–T04, T07, T11–T13) exhibit low values (Figs. 3 and 4A and Supplementary Table 1).

(ii) *Selecting vertebrae by position*: Although using this approach, there is a slight decrease in the MDI values obtained for Tmid, Diaph, Tlast and Llast, the pattern of MDI distribution obtained is very similar to the one obtained grouping vertebrae by number (Fig. 3 and Supplementary Table 1). Disparity through time curves consistently shows a high proportional subclade disparity through most of the evolutionary history of Carnivora (Fig. 4B and Supplementary Figure 1). A decrease in MDI from the Tfirst to the Tmid-Diaph and subsequent increase to Tlast-Lfirst is noticed. Finally, there is a decrease in MDI values from Lfirst to Lmid-Llast.

### Influence of ecology on vertebral morphology

**Fig. 5:**
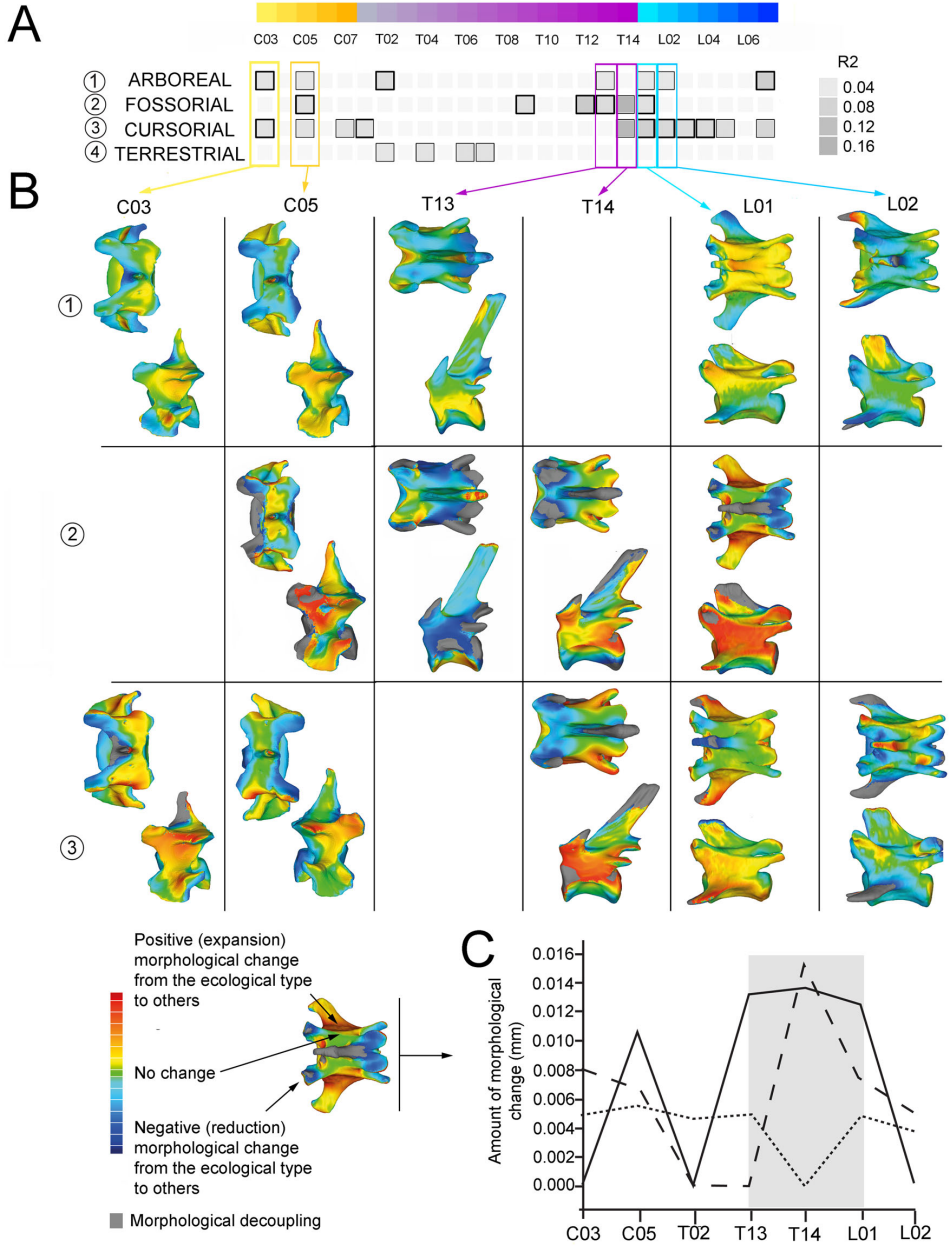
Ecomorphological analysis of presacral vertebra shapes excluding aquatic taxa and grouping vertebrae by number. **A** Vertebrae significantly associated with a given ecological category (1, arboreal; 2, fossorial; 3, cursorial; 4, terrestrial). The significance of *R*^2^ (grey-colour squares) for each vertebra is indicated by frame thickness; thick frames *p* values <0.01; thin frames *p* values <0.05; absence of frame indicates *p* values >0.05. **B** Three-dimensional models obtained from phylogenetic Procrustes ANOVAs showing for each for each significant association of “vertebra-ecology” the morphological deviation from the corresponding shape for a given ecology (red) to the vertebral shape of the rest of the sample (blue). Note that only some vertebrae for each region (cervical, anterior thoracics, posterior thoracics and lumbars) are shown. **C** Quantification of the morphological deviation obtained from the three-dimensional models represented in (**B**) showing that most of the change occurs in the posterior thoracics and lumbars. Dotted line represents morphological deviations for arboreality, dashed line for cursoriality, and bold line for fossoriality.

(i) *Grouping vertebrae by number*: Procrustes ANOVAs indicate a strong effect of aquatic capability on vertebral morphology (Supplementary Figure 2, Supplementary Table 1, Supplementary Data 1 and Supplementary Data 2) and weaker effects for other locomotor capabilities (cursorial, fossorial, arboreal, terrestrial; Fig. 5A, Supplementary Table 1, Supplementary Data 1, Supplementary Data 1 and Supplementary Data 2). Accordingly, aquatic capability exhibits a significant effect (*p* < 0.05) on all elements of the vertebral column with *R*^2^ up to 0.29 (Supplementary Table 1). This occurs even when excluding pinnipeds from the analysis. The large effect size of aquatic capability on vertebral morphology reduces the apparent influence of other locomotor capabilities, which are stronger and more significant when excluding aquatic taxa than when including them.

**Fig. 6:**
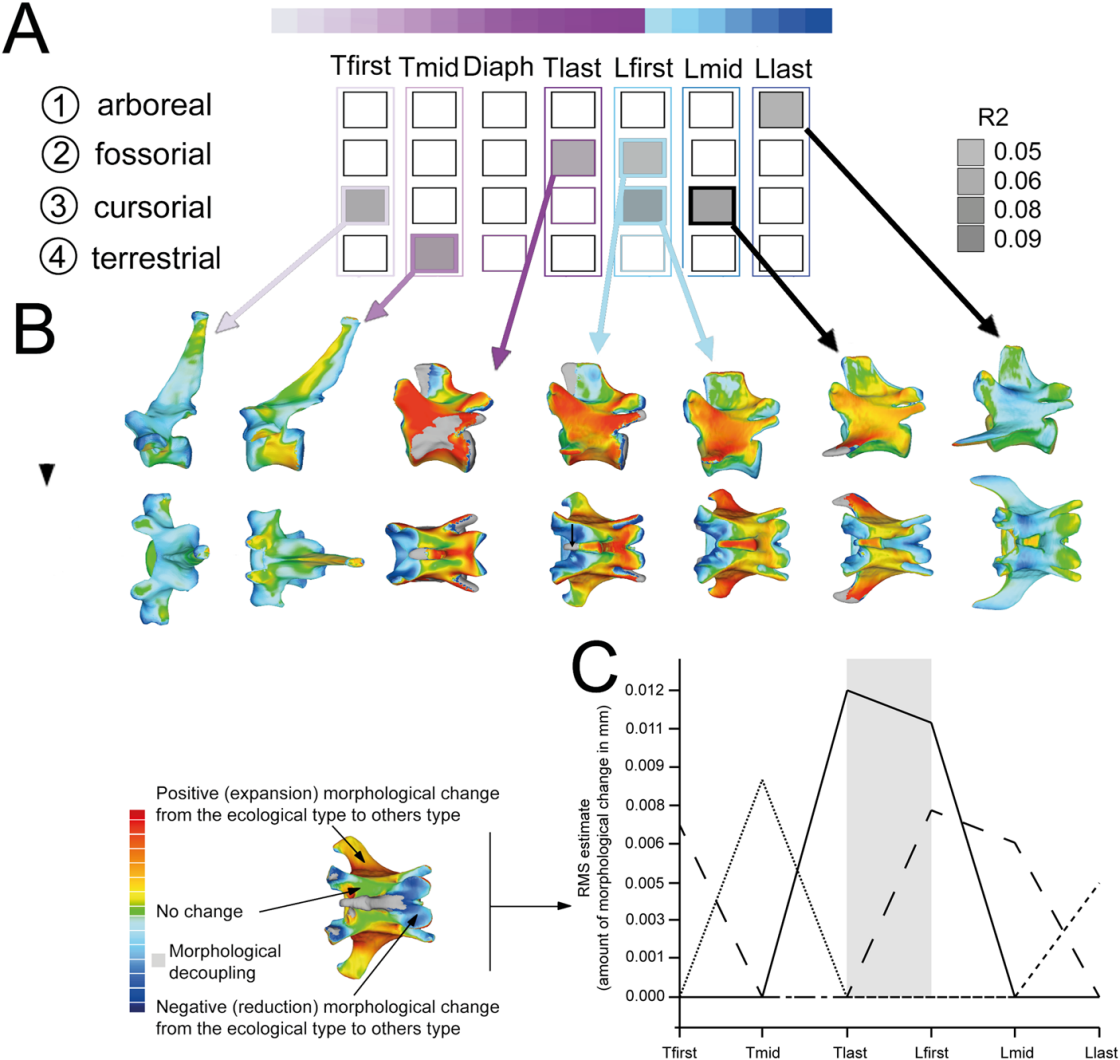
Ecomorphological analysis of presacral vertebra shapes excluding aquatic taxa and selecting vertebrae by position. **A** Vertebrae significantly associated with a given ecological category (1, arboreal; 2, fossorial; 3, cursorial; 4, terrestrial). The significance of *R*^2^ (grey-colour squares) for each vertebra is indicated by frame thickness; thick frames *p* values <0.01; thin frames *p* values <0.05; absence of frame indicates *p* values >0.05. **B** Three-dimensional models obtained from phylogenetic Procrustes ANOVAs showing for each for each significant association of “vertebra-ecology” the morphological deviation from the corresponding shape for a given ecology (red) to the vertebral shape of the rest of the sample (blue). **C** Quantification of the morphological deviation obtained from the three-dimensional models represented in (**B**) showing the vertebrae that experience most of the changes in shape. Dotted line represents morphological deviations for arboreality, dashed line for cursoriality, and bold line for fossoriality.

Our analyses excluding aquatic taxa show that the other locomotor capabilities are associated with morphological differences among different regions of the vertebral column (Fig. 5A and Supplementary Data 3). Habitual terrestrial capability has the weakest effect, influencing the morphology of some thoracic vertebrae only (T02, T04, T06 and T07), and with marginal significance (*p* < 0.1; Fig. 5A and Supplementary Table 1). In contrast, arboreal (*p* < 0.05 in the C03, T02 and L07), fossorial (C05, T09, T12, T13, T14 and L01) and cursorial (C03, T01 and L01–L04) capabilities influence the shapes of more vertebrae, and with stronger significance (Fig. 5A). Higher *R*^2^ values for these groupings are mainly concentrated across the thoracic–lumbar transition, although at different positions. For example, arboreality influences the shapes of the T13–L02 and L07 (*p* < 0.1 in at least some analyses; Fig. 5A) fossoriality influences the shapes of T07, T09 and T12–L02 (*p* < 0.1 in at least some analyses), and cursoriality influences the shapes of T14–L05 (*p* < 0.1 in at least some analyses). The morphological deviations of each ecomorphological type (i.e. arboreal, terrestrial, cursorial, fossorial) from the rest of the sample for those vertebrae whose shape was significatively associated with a particular locomotor adaptation are shown in Fig. 5B.

(ii) *Selecting vertebrae by position*: As in the analysis performed grouping by vertebra, Procrustes ANOVAs indicate a strong effect of aquatic capability on vertebral morphology (Supplementary Figure 3, Supplementary Table 1 and Supplementary Data 2) and weaker effects for other locomotor capabilities (Fig. 6A and Supplementary Data 2). Accordingly, aquatic capability exhibits a significant effect (*p* < 0.05) on all elements of the vertebral column (Supplementary Table 1). Again, the exclusion of aquatic taxa increases the apparent influence of other locomotor capabilities.

Locomotor capabilities are associated with morphological differences among thoracic and lumbar regions of the vertebral column (Fig. 6A and Supplementary Data 3). Terrestrial and arboreal capabilities have the weakest effect, influencing the morphology of only one vertebra, Tmid and Llast, respectively, and with marginal significance for the arboreals (Fig. 6A and Supplementary Table 1). In contrast, fossorial and cursorial capabilities influence the shapes of more vertebrae: Tlast and Lfirst for fossorial capabilities; Tfirst, Lfirst and Lmid for cursorials, and with strong significance (Fig. 6A).

The morphological deviations of each ecomorphological type (i.e. arboreal, terrestrial, cursorial, fossorial) from the rest of the sample for those vertebrae whose shape was significatively associated with a particular locomotor adaptation are shown in Fig. 6B.

### Statistical comparisons

**Fig. 7:**
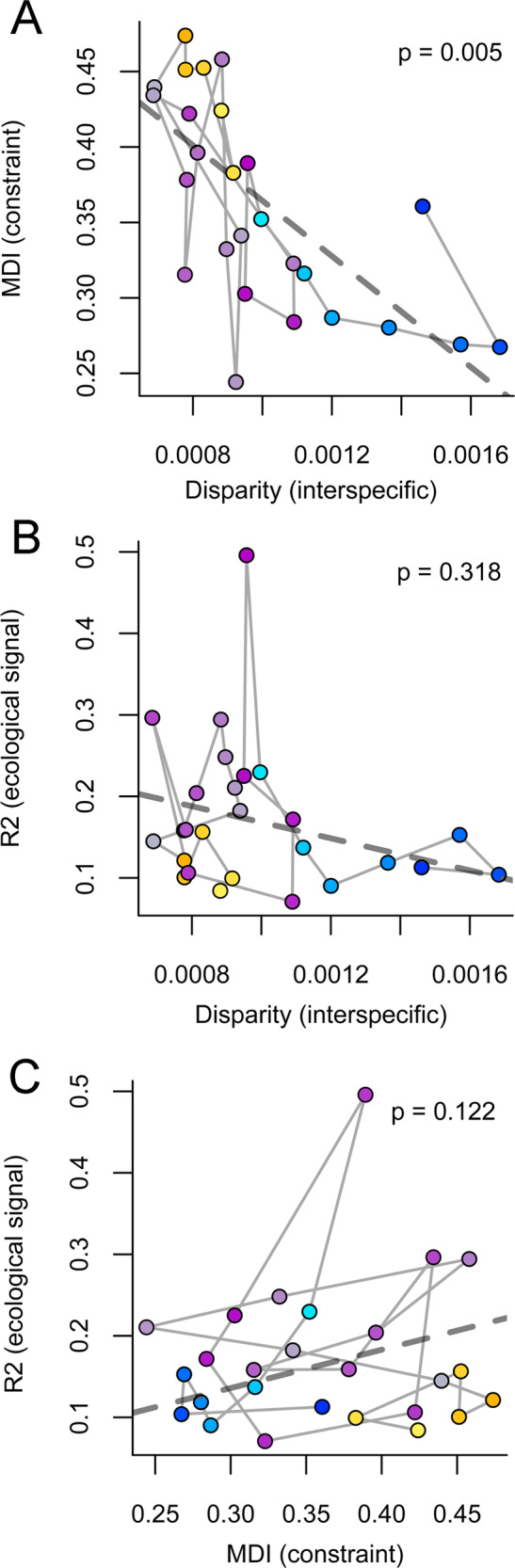
Relationships between ecomorphological adaptation, evolutionary constraint (MDI) and among-species disparity grouping vertebrae by number. **A** Bivariate plot of MDI values against among-species disparity. **B** Bivariate plot between the strength of ecomorphological signal (*R*^2^ from the best Procrustes ANOVA analyses) and interspecific disparity. **C** Bivariate plot between the strength of ecomorphological signal (*R*^2^ from the best Procrustes ANOVA analyses) and evolutionary constraint (MDI).

**Fig. 8:**
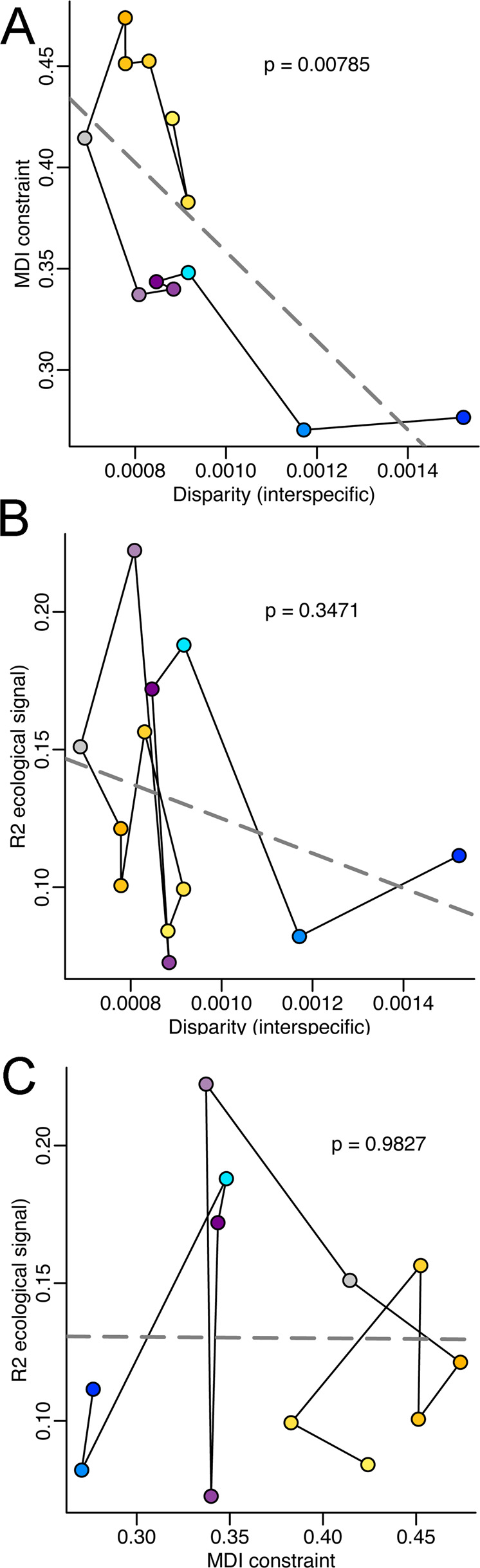
Relationships between ecomorphological adaptation, evolutionary constraint (MDI) and among-species disparity selecting vertebrae by position. **A** Bivariate plot of MDI values against among-species disparity. **B** Bivariate plot between the strength of ecomorphological signal (*R*^2^ from the best Procrustes ANOVA analyses) and interspecific disparity. **C** Bivariate plot between the strength of ecomorphological signal (*R*^2^ from the best Procrustes ANOVA analyses) and evolutionary constraint (MDI).

(i) *Grouping vertebrae by number*: Generalised least-squares regressions indicate a statistically significant inverse correlation between interspecific disparity and MDI (Fig. 7A, *p* = 0.005, *N* = 26 vertebral positions, Supplementary Table 1), indicating that low constraint is in general associated with high disparity. Deviations from this relationship occur in the anterior-mid thoracic region (Figs. 3 and 6). Relationships between the strength of ecomorphological signal (*R*^2^ from the best Procrustes ANOVA analyses) and disparity (Fig. 7B; *p* = 0.318) or MDI (Fig. 7C; *p* = 0.122) are consistently non-significant.

(ii) *Selecting vertebrae by position*: Similarly, using this approach, a statistically significant inverse correlation between interspecific disparity and MDI (Fig. 8A; *p* = 0.00785, Supplementary Table 1), indicating that low constraint is in general associated with high disparity. The relationships between the strength of ecomorphological signal (*R*^2^ from the best Procrustes ANOVA analyses) and disparity (Fig. 8B; *p* = 0.3471) or MDI (Fig. 8C; *p* = 0.9827) are consistently non-significant.

## Discussion

Grouping vertebrae by number, we find that the total size of vertebral morphospaces (i.e. morphological disparity) is related to the mode of trait space exploration by evolving lineages. As expected, vertebrae with more constrained patterns of evolution have low disparities, even though constraint—the extent to which evolving lineages have repeatedly explored the same set of morphologies—is quantified without reference to the amount of difference among those morphologies (i.e. disparity; Fig. 7A). Moreover, the approach of selecting vertebrae by position, and hence ensuring the homology for some of the comparisons, confirms the significant inverse relationship between disparity and constraint (Fig. 8A).

We also find that locomotory adaptation, especially adaptation to aquatic locomotion, has a strong influence on the vertebral form using both sampling schemes (Supplementary Figure 2, Supplementary Figure 3 and Supplementary Table 1). Nevertheless, these results demonstrate that the strength of this ecomorphological signal does not have a predictable influence on either disparity (Figs. 7B and 8B) or the apparent strength of constraint (Figs. 7C and 8C)—although ecologies besides locomotion may also be important. This suggests that ecomorphological adaptation plays a complex role in structuring the evolution of the vertebral column.

Evolution of the carnivoran presacral column is generally highly constrained. Disparity through time curves for each vertebra consistently show a high proportional subclade disparity through most of the evolutionary history of Carnivora (Fig. 4 and Supplementary Figure 1). This is consistent with the observation that even relatively young subclades have highly overlapping distributions in vertebral morphospace. Cervicals have the highest MDI values among all presacral vertebrae, indicating proportionally greater constraint on cervical evolution than on vertebrae of other regions (Fig. 3 and Table 1). Grouping vertebrae by number indicate that lumbars have MDI values within the range of variation of the thoracics, but many thoracics exhibit higher values than any lumbar vertebra. Selecting vertebrae by position indicates that while the last and intermediate lumbars have significantly lower MDI values than any thoracic, the first lumbar exhibits higher MDI values than other lumbars and comparable to the last thoracic. This suggests that lumbars and some thoracics exhibit relatively less-constrained evolution than other vertebrae. These patterns are inverse of disparity (Figs. 3, 7A and 8A), and also contrast to some extent with patterns of serial differentiation: lumbars are not as well differentiated according to their position (i.e. adjacent vertebrae have similar morphologies), whereas cervical morphologies show much greater differentiation (Fig. 2). Moreover, these patterns of interspecific disparity and MDI do not result from their artificial inflation due to variation in count among species.

Our results indicate that aquatic adaptation has a particularly strong effect on the morphology of the whole axial column (Supplementary Figures 2 and 3). In contrast, the signals of other locomotor capabilities are most prominent in posterior thoracics and lumbars (Figs. 5 and 6), supporting previous findings for the family Felidae^37–39^ and for mammals^13^. The relationships between vertebral form and locomotor function are statistically supported by Procrustes ANOVA and show generally high *R*^2^ values indicating that lumbar and thoracic vertebrae are recruited into the locomotory function (Figs. 5A and 6A and Table 1). Locomotor-related shape changes of posterior thoracic and lumbar vertebrae are varied, involving changes to the length and width of the vertebrae but also the orientation and disposition of different processes and facets (Figs. 5 and 6). Grouping vertebrae by number, the identities of vertebrae that reflect adaptation to each ecological grouping vary in number (i.e. for arboreality [for T13, L01, L02 and L07], for fossoriality [T09, T12–L01] and for cursoriality [T14–L05, L07]). This suggests that the diaphragmatic “region” reported by Randau and Goswami^35^ in felids (and broadly across mammals by Jones et al.^13^) is widespread among carnivorans, and represents an overlapping and non-mutually exclusive set of a functional subset of vertebrae exhibiting independent ecomorphological responses to locomotor evolution.

A similar result is obtained selecting vertebrae by position, as the identities of vertebrae that reflect adaptation to each ecological grouping vary in position (i.e. for arboreality [Llast], for fossoriality [Tlast, Lfirst], for cursoriality [Lfirst, Lmid]) (Fig. 6). This pattern is very similar to the one obtained by grouping vertebrae by number (Fig. 5), as the only difference among both approaches is that those vertebrae with very weak ecological signals grouping by number result as non-significant when selecting vertebrae by position. This is the case for the slightly significant T13–L02 for arboreal capabilities grouping vertebrae by number (Fig. 5), which result in not significant selecting vertebrae by position (Fig. 6). Indeed, only the last lumbar (Llast) results as significant selecting vertebrae by position, which is the vertebrae with the highest ecological signal within arboreality (note that T02 is lacking in the second approach). Similarly, for cursorial capabilities, the very weak ecological signal of L07 (Fig. 5) is blurred when selecting vertebra by position (Fig. 6). Despite this, other significant vertebrae include the first thoracic (Tfirst), as well as the first (Lfirst) and intermediate lumbars (Lmid) selecting by position (Fig. 6), which coincides with those vertebrae with higher ecological signals grouping by number (Fig. 5). For fossoriality the last thoracics and first lumbars result as significant with both approaches, as T09 is lacking in the approach of selecting vertebrae by position (Figs. 5 and 6). Similarly, terrestrial capabilities only recruit those vertebrae for the intermediate thoracic region using both approaches (T02, T04, T06–T07 and Tmid) (Figs. 5 and 6).

Other vertebrae are also recruited into distinct locomotory functions, but nevertheless show different macroevolutionary dynamics to the posterior thoracics and lumbars. These include C03, C05 and T02 (or Tfirst, selecting vertebrae by position; see Fig. 5A). Unlike the posterior thoracics and lumbars, these vertebrae have low interspecific disparity and high MDI values—i.e. small overall morphospace and constrained patterns in which evolution has repeatedly explored a limited distribution of morphologies. Vertebrae from these regions also differ from those of the posterior thoracic and lumbar vertebrae in showing greater morphological differentiation from adjacent vertebrae (Fig. 2), which may reflect greater functional differentiation of these vertebrae due to their roles in functional tasks other than locomotion (e.g. forelimb and head mobility as well as rib attachment)^35^. Consistent with this hypothesis, locomotor-related evolutionary variation may have modified these vertebrae within fewer dimensions than for the posterior thoracics and lumbars: C03, C05 and T02 (among others; Supplementary Fig. 1) change broadly along the same morphological axes in response to all locomotory capabilities (Figs. 5B and 6B; changes in the length and width of the vertebra).

The patterns of ecomorphology suggest that the evolution of form–function relationships of the cervicals and anterior thoracics is explained by a one-to-many mapping (i.e. the same morphological solution to meet several adaptive challenges) pattern of evolution, while the evolution of posterior thoracics and lumbars accords with a pattern of a one-to-one mapping (i.e. one morphological solution to each adaptive challenge). This may result from the functional role of the lumbar region (and posterior thoracics, to a lesser degree), which is almost exclusively involved in locomotion (i.e. hindlimb and vertebral column mobility) and hence having more flexibility for morphological change towards a specific locomotor strategy. This is supported by kinematic analyses, as the extent of sagittal bending during running varies among species, encompassing lumbars and posterior thoracics in some species but restricted to the lumbar region in others^13,37^.

Our findings are consistent with the hypothesis that the lumbar region of therians represents an evolutionary innovation that enables high evolvability and ecological plasticity^12,14^. We hypothesise that functional specialisation for locomotion in these vertebrae (posterior thoracics and lumbars) gives rise to this pattern, in which adaptation is correlated with high disparity and relaxed constraints. In contrast, the constrained morphology of cervicals and anterior thoracics restrains their disparity, and limits the dimensionality of their form–function responses. Nevertheless, the occurrence of constraints on the evolution of these anterior vertebral units does not altogether precludes the occurrence of ecomorphological adaptations towards specific locomotory demands. This indicates that the strength of ecomorphological signal does not have a predictable influence on macroevolutionary outcomes—as indexed by either disparity and constraint (or vice versa), a finding that undermines the traditional view that highly constrained skeletal units are strongly limited in their potential to adapt to new ecological functions.

The approach “grouping vertebrae by number” developed here allows the analysis of both local-scale (within regions) and broad-scale (among regions) morphological variations, which warrants the possibility of quantifying the influence of ecomorphological adaptation on macroevolutionary outcomes as indexed by either disparity or constraint. However, this could entail problems establishing homology. The homology of vertebrae is established from the homology of *Hox* genes expressed at each level^36,38^. For example, the anterior expression boundary of *HoxC6* falls at the same morphological boundary between the cervical and thoracic region in mouse and chicken, irrespective of lying the boundary at different axial levels and the segmental number of somites (i.e. while the seventh vertebra is the last cervical one in the mouse, the 14th is the last cervical in the chicken)^36,38^. Accordingly, grouping vertebrae by number conflates variation from different types of vertebrae from some of the comparisons (i.e. posterior thoracics and first lumbars) and may ignore within-region homology. For example, there is consistent variation along the vertebral column in mammals such that vertebrae from the anterior thorax differ from the posterior thorax, and those from the anterior lumbus differ from the posterior lumbus, as well as varying among regions. Therefore, the exact position of a given vertebra within a particular region is extremely important for determining its morphology, irrespective of the number of vertebrae in that region.

To solve this problem, we followed the approach of Jones et al.^13^ and we selected some vertebrae (by position) with, a priori, clearer homologous criteria between taxa with different counts, instead of selecting taxa with comparable vertebral formulas. We avoid the latter to maximise sample size, as some key taxa (e.g. hyaenids or ursids) depart from the 13 T + 7 L, and they could not be included in the analyses. Selecting vertebrae by position solves the problem of conflating the variation resulting from the comparison of different types of vertebrae, and hence avoiding the comparison of a last lumbar in one species (e.g. L04 in a species with solely four lumbars) with a middle lumbar in another (e.g. L04 in a species with seven lumbars). However, a weak point of this sampling scheme is the assumption that intermediate thoracics or lumbars among taxa with different counts are considered homologous, and this implies that vertebrae are added or deleted from one taxon to another in an equal fashion along the column. Moreover, this method lacks the perspective of local-scale mophological variation, and the few numbers of vertebrae that could be matched across all the sample with criteria based on anatomy or position prevents to perform robust statistical testing.

Our recommendation to perform cross-taxonomic studies including taxa with different counts is to perform both types of analyses and taking careful consideration about areas of differential results. The first analysis “grouping vertebrae by number” provides the perspective of both local-scale (within regions) and broad-scale (between-regions) variations and it allows to explore further statistical analyses for which larger samples are required. However, special attention should be paid to those comparisons between less-clear homologous subunits. On the other hand, the second analysis “selecting vertebrae by position” warrants that most of the comparisons are performed between homologous vertebrae.

Overall, both analyses presented here clearly give the same results except for exceptional cases. For example, the marked difference between the disparity at L07 and that at the last lumbar position (Tlast). However, this is probably due to sampling differences, as while all 44 species have a Tlast, only 22 species have L07. Strikingly, the disparity values obtained for the other six selected vertebrae by position overall match with the pattern of disparity found grouping vertebrae by number, which clearly confirms that our results concerning disparity and constraint are robust.

Another point of disagreement between both analyses is the number of vertebrae with significant ecological signals. Overall, the ecological signal decreases selecting vertebrae by position relative to grouping vertebrae by number, and this is especially evident in those vertebrae that exhibit very low signals grouping by number, and therefore their correlation with ecology should be interpreted with caution.

The results obtained from both sampling schemes developed here indicate that patterns of ecomorphological adaptation and evolutionary constraints are more complex than previously recognised. Functional trade-offs are often viewed as imposing constraints on phenotypic evolution but might also facilitate evolution across the suboptimal valleys separating performance peaks on performance landscapes^39^. Our findings of low-dimensional ecomorphological variation in constrained elements of the carnivoran vertebral column provide heuristic support for this hypothesis. In this case, the one-to-many pattern where the same phenotype can accomplish different adaptive challenges seems to explain the evolution of these constrained elements.

The study of phenotypes hierarchically organised below the static level—within species at an adult stage—such as that presented here, emphasise the necessity to analyse both local- and broad-scale morphological variation in multi-element and functionally versatile systems without overlooking homology among the subunits compared.

## Methods

### Data collection

All presacral vertebrae excluding the first and second cervical vertebrae (atlas and axis, respectively) of 44 species of mammalian carnivores (Supplementary Data 1) were scanned in 3D with either micro-computed tomography (CT), regular CT scanning or a NextEngine® surface scanner. CT scans were segmented in Avizo. This resulted in 3D models of 1097 vertebrae. The 3D models were imported into Meshlab^40^ to reduce the size of the models.

**Fig. 9:**
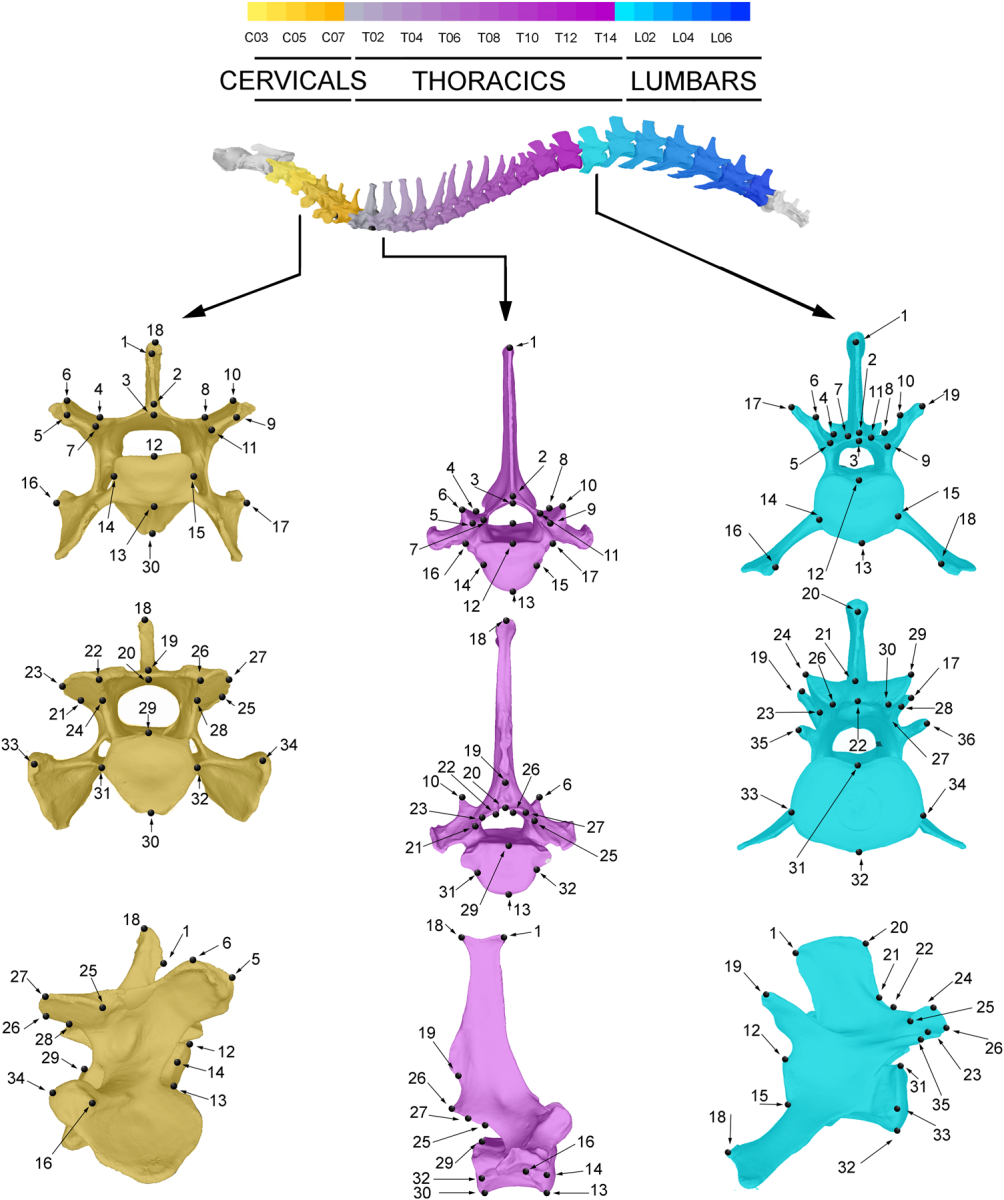
Landmarks digitized in this study, taking the 3D models of a third cervical, first thoracic and fifth lumbar vertebra of a grey wolf (*C. lupus*) as an example. For information on the anatomical criteria followed to digitise de landmarks see Supplementary Table 2.

To capture the morphology of the vertebrae, we digitized 34 homologous landmarks on the cervical vertebrae (C03–C07), 32 homologous landmarks on the thoracic vertebrae (T01–T14), and 36 homologous landmarks on the lumbar vertebrae (L1–L7) (see Fig. 9, Supplementary Table 2 and Supplementary Data 4). The landmarks were digitized with the software *Landmark* from IDAV^41^ and the *x*, *y*, *z* coordinates of each landmark were exported as a Text file. Although a set of landmarks could easily be determined across all of the elements, we digitized a different number of landmarks in each region, because disparity analyses are important to recover as much morphological information as possible.

Raw landmark data were imported into the R package *geomorph* version 3.1.0^42^. Each region of the vertebral column (cervical, thoracic and lumbar) was separated into different datasets, and therefore all subsequent analyses were performed independently.

We performed a Procrustes alignment for each region taking into account the bilateral symmetry of the vertebrae using the function *gpagen* and *bilat.symmetry*, respectively. Afterwards, we averaged the landmarks for each vertebra of those species that were represented by more than one individual to minimise the effects of intraspecific shape variation. Our phylogeny-based analyses^43^ used the phylogenetic framework of Nyakatura and Bininda-Edmonds^44^ (Fig. 1).

### Statistics and reproducibility

(1) *Disparity calculations and PCA*: to explore the morphological variability of the vertebral column, we performed a PCA for each region using the function *plotTangentSpace*. For easier visualisation, a 3D surface model was incorporated into R using the function *read.ply* and transformed according to the shape changes associated with the two first PCs (PC1 and PC2) obtained using the functions *mshape* and *plotRefToTarget* from *geomorph*.

Once the data were explored with a classic ordination analysis such as PCA, we further subdivided our dataset following two different sampling schemes to perform interspecific analyses: (1) grouping vertebrae by number; and (2) selecting a subset of vertebrae selected by position or anatomical relevance irrespective of count (e.g. joining the last lumbar of across taxa irrespective of being the L05 in one taxon and the L07 in another). To do this, we have selected some key vertebrae according to their position or anatomical relevance for which homology is clearer. These are the first, intermediate (T06 for species with 13 or 14 thoracics and T07 for species with 15 or 16 thoracics) and last thoracics (named as Tfirst, Tmid and Tlast, respectively), the diaphragmatic vertebra (Tdiaph) and the first, intermediate (L03 for species with four and five lumbars and L04 for species with six or seven lumbars) and last lumbars (Lfirst, Lmid and Llast). The cervicals are also considered in this sampling scheme but they do not differ from the approach selecting vertebrae by number, as all the taxa have a fixed cervical count at seven (as almost all mammals).

We computed morphological disparity (Procrustes variance)^45^ for each region using the function *morphol.disparity*. We calculated vertebral disparity, grouping by vertebrae by number within each region (e.g. all C03, or all T14, or all L07 present in our dataset in order were grouped together) and selecting vertebrae by position (i.e. Tfirst, Tmid, Tlast, Tdiaph, Lfirst, Lmid and Llast) to assess the disparity among species for each vertebra (hereafter, interspecific disparity). Grouping vertebrae by number, as all the species have the same number of cervicals and, at least, 13 thoracics, the analyses of these vertebrae (C03–T13) were computed including all taxa. However, the analyses of T14 and T15 were restricted to only the taxa that have 14 or 15 thoracics, respectively. Similarly, all the taxa have at least four lumbars, but the analyses for L05, L06 and L07 were restricted to those taxa that have five, six or seven lumbars, respectively. It should be noted that meristic variation is almost absent in our dataset (all species have 27 presacral vertebrae with the only exception of *Ailuropoda melanoleuca* [see Fig. 1]), and the variation is mostly homeotic. The only problem using the sampling scheme grouping vertebrae by number is that we have variability in sampling for five vertebrae (T14, T15, L05, L06 and L07).

In both cases, as each region shares a specific set of digitized landmarks, to compare among-region disparities, we standardised disparity values by the number of landmarks (disparity/number of landmarks). Three Procrustes ANOVAs were performed for each region using the *procD.lm* function^46^, using vertebral identity as a grouping factor. This function computes the effect size, *Z*-score, which quantifies the amount of morphological variability explained by the grouping factor.

(2) *Disparity through time analyses*: For each vertebra, we calculated the proportional disparities of carnivoran subclades in relation to total carnivoran disparity, and how this varied through time (i.e. for subclades of different ages) using the “disparity through time” method of Harmon et al.^47^ applied to allometry-corrected vertebral shape data (i.e. the residuals of a phylogenetic Procrustes ANOVA of shape on size^48^) for both sampling approaches—i.e. grouping by number and with a set of selected vertebrae by position. This is implemented in the *dtt* function of the R package *geiger*^48^. This describes the timing of evolutionary morphospace partitioning among subclades and is compared to an expectation line computed by simulating continuous multivariate trait evolution under Brownian motion^49^. High relative subclade disparities indicate that subclades have highly overlapping morphospaces and high disparity, expressed as a share of the whole-clade disparity. This suggests constrained morphological evolution in which subclades repeatedly explore the same regions of morphospace. Low relative subclade disparities indicate strong partitioning of morphospace into clade-specific subspaces that each occupies a small proportion of the whole-clade disparity. This variation is summarised using the MDI, which describes the difference between the observed DTT trajectory and the expected DTT trajectory according to a BM model, using the R package geiger version 2.0.6.1^49,50^. Positive MDI values indicate substantial morphological overlap between subclades, whereas negative MDI values indicate that subclades have individually low disparities compared to the total clade, suggesting partitioning of morphospace among subclades^51^.

(3) *Correlations of vertebral shape vs locomotor capabilities*: To test the association between vertebral shape and the ecological capabilities of species, we used a phylogenetic Procrustes ANOVA for each vertebra using the procD.pgls function^46,48^, with a set of variables that define the ecology of the species as covariates. We used a modified version of the ecological classification of Samuels et al.^52^, with additional information from Wilson and Mittermeier^53^ for taxa that otherwise would not be included. Whereas most previous studies of mammalian ecomorphology assign each species to a single ecological category (e.g. terrestrial | cursorial | arboreal | scansorial | semi-aquatic), we use a multivariate presence/absence classification that recognises the broad locomotory capabilities of each taxon (Table S4). This is similar to the multivariate categorisation of flight capabilities proposed by Taylor and Thomas^54^ and allows tests of the relationship between individual locomotor capabilities and vertebral morphologies.

Within this multivariate framework, “terrestrial” capability is scored as present in all species that habitually walk on the ground, including taxa coded as terrestrial, cursorial, scansorial, semi-aquatic or semi-fossorial in previous classifications. “Cursorial” capabilities are scored as present in all carnivorans whose forelimbs are used primarily for terrestrial locomotion, including long-distance pursuit hunters that do not extensively grapple with prey (i.e. many canids, hyaenids and the cheetah among felids), following Figueirido et al^55^. Therefore, the maned wolf (*Chrysocyon brachyurus*) was classified as a cursor in our study (unlike in Samuels et al.^52^) because it behaves in a similar fashion to any other fox and does not manipulate items nor grapples with prey^56–58^, using the forelimb primarily for terrestrial locomotion following^59^. “Arboreal” capability is scored as present in species coded as arboreal or scansorial in previous classifications. “Aquatic” capability is scored as present in species coded as aquatic or semi-aquatic in previous classifications—with the presence (in e.g. *Lutra lutra*) or absence (in pinnipeds) of habitual terrestrial capability distinguishing between these traditional classifications. “Fossorial” capability is scored as present in all taxa that habitually use the forelimb to excavate, including species that were scored as semi-fossorial in previous classifications (e.g. *Meles meles*, *Suricatta suricatta*) as well as some taxa that were more typically scored as terrestrial (e.g. *Herpestes ichneumon*).

Our phylogenetic Procrustes ANOVA analyses ask what proportion of variance in the allometry-corrected vertebral shapes (i.e. residuals from phylogenetic Procrustes ANOVA of shape on size) is explained by each ecological capability, and also constructs the best model that can be made for each vertebra using multiple explanatory variables. We evaluated every combination of explanatory (ecological) variables, calculating *R*^2^ based on the hierarchical sum of squares, and selected the model with the highest *R*^2^ from the set of models in which all explanatory variables had at least marginal statistical significance (*p* < 0.1). This *R*^2^ value represents the overall strength of ecomorphological adaptation across all significant or marginally non-significant locomotory variables.

Comparison of these models provides a direct test of the extent to which each locomotory capability explains variation in vertebral shape. For example, scansorial taxa might share vertebral traits either with arboreal taxa, terrestrial taxa or both. In our classification, they are scored as “1” for both arboreality and terrestriality, and our model comparisons provide independent tests of both effects. Similarly, all cursorial taxa are also terrestrial, and shared shape features might be present in both “cursorial” and “terrestrial” species, arising from their terrestrial habits, a hypothesis that is tested by our analyses.

The ecological analyses were performed from both sampling schemes—i.e. grouping by number and selecting a set of vertebrae by position.

### Statistical comparisons

We examined the correlations among disparity, MDI (constraint) and the strength of ecomorphological signal among vertebral positions using generalised least-squares linear regressions implemented in the R package nlme version 3.1-139^60^. These analyses incorporated a first-order autoregressive model^61^ to account for serial correlation of adjacent vertebral morphologies along the column, and were performed with two independent datasets, grouping by number and selecting a set of vertebrae by position.

### Reporting summary

Further information on research design is available in the Nature Research Reporting Summary linked to this article.

## Supplementary information

Supplementary Information

Description of Additional Supplementary Files

Supplementary Data 1

Supplementary Data 2

Supplementary Data 3

Supplementary Data 4

Reporting Summary

## Data Availability

The data are uploaded as Supplementary information.
