# Supplementary material for: Serial disparity in the carnivoran backbone unveils a complex adaptive role in metameric evolution
Source: Commun Biol. 2021 Jul 15;4:863. doi: 10.1038/s42003-021-02346-0 (PMC8282787; doi:10.1038/s42003-021-02346-0)

SUPPLEMENTARY INFORMATION

Supplementary Table 1. Average values of MDI, disparity (among-species), and the coeffcient R2 obtained from the association between vertebral shape and ecology for the total sample and the subsample of selected vertebrae.

	R2_ecology	MDI_constraint	Disparity_interspecific
C03	0.084118402	0.424126014	0.000881802
C04	0.099325304	0.382853701	0.000916322
C05	0.156418774	0.452461336	0.000830649
C06	0.100617224	0.451221723	0.000778625
C07	0.121279142	0.473798005	0.000778288
T01	0.144846326	0.439613068	0.000689175
T02	0.182277209	0.341222736	0.000939032
T03	0.210469698	0.24420661	0.000923965
T04	0.24811591	0.332280782	0.000896717
T05	0.294258905	0.458048688	0.000883634
T06	0.204012486	0.396210157	0.000813306
T07	0.158227272	0.31533865	0.000776998
T08	0.158991758	0.378255735	0.000782597
T09	0.296418926	0.434294488	0.000686145
T10	0.106160329	0.422037185	0.000789416
T11	0.07074495	0.32275317	0.001089321
T12	0.171719779	0.283972463	0.0010911
T13	0.22510571	0.302667741	0.000949968
T14	0.495841339	0.389282946	0.000957385
L01	0.229596556	0.3521718	0.000996552
L02	0.137139904	0.316128931	0.001120532
L03	0.090195907	0.286780743	0.00120036
L04	0.118574677	0.280284625	0.001364222
L05	0.152730923	0.269131513	0.001570679
L06	0.103870753	0.267377247	0.001683677
L07	0.112842173	0.360612499	0.001461222
Tfirst	0.15102007	0.4290115	0.0006891749
Tmid	0.22228539	0.3339933	0.0008084856
Diaph	0.07265157	0.3288350	0.0008845713
Tlast	0.17197223	0.3615466	0.0008468083
Lfirst	0.18795103	0.3639143	0.0009169951
Lmid	0.08212667	0.2596935	0.0011710842
Llast	0.111493	0.2685011	0.0015223624

Supplementary Table 2. Number of landmarks digitized and the anatomical criteria per region used in this study. See also Figure 2.

REGION	LANDMARKS	DEFINITION
Cervical	1-2	cranial **spinous process**
	3	cranial mid-point **neural canal**
	4-7	cranial left **articular process**
	8-11	cranial right **articular process**
	12-15	cranial **body**
	16	cranial left **transverse process**
	17	cranial right **transverse process**
	18-19	caudal **spinous process**
	20	caudal mid-point **neural canal**
	21-24	caudal left **articular process**
	25-28	caudal right **articular process**
	29-32	caudal **body**
	33	caudal left **transverse process**
	34	caudal right **transverse process**
Thoracic	1-2	cranial **spinous process**
	3	cranial mid-point **neural canal**
	4-7	left cranial **articular process**
	8-11	right cranial **articular process**
	12-15	cranial **body**
	16	left cranial **costal facet**
	17	right cranial **costal facet**
	18-19	caudal **spinous process**
	20	caudal mid-point **neural canal**
	21-24	left caudal **articular process**
	25-28	right caudal **articular process**
	29-32	caudal **body**
Lumbar	1-2	cranial **spinous process**
	3	cranial mid-point **neural canal**
	4-7	left cranial **articular process**
	8-11	right cranial **articular process**
	12-15	cranial **body**
	16	left cranial **transverse process**
	17	left **mamillary process**
	18	right cranial **transverse process**
	19	right **mamillary process**
	20-21	caudal **spinous process**
	22	caudal mid-point **neural canal**
	23-26	left caudal **articular process**
	27-30	right caudal **articular process**
	31-34	caudal **body**
	35	left caudal **transverse process**
	36	right caudal **transverse process**

Supplementary Figure 1. Disparity-through-time (DTT) plots for vertebral shape using the residuals from allometric regression (procD.lm of vertebral shape on vertebral centroid size) arranged by region: cervicals (A), thoracics (B) and lumbars (C). The Y axis is the average subclade disparity expressed as a proportion of total clade for subclades originating at different times (X axis). Relative time values are used with 0.0 representing the root age and 1.0 representing the present. The solid line indicates the mean DTT from the empirical dataset, and the dashed line the simulated datasets. Dashed lines indicates the 95% DTT range (grey) and central tendency (black) for the simulated multivariate data under Brownian motion.

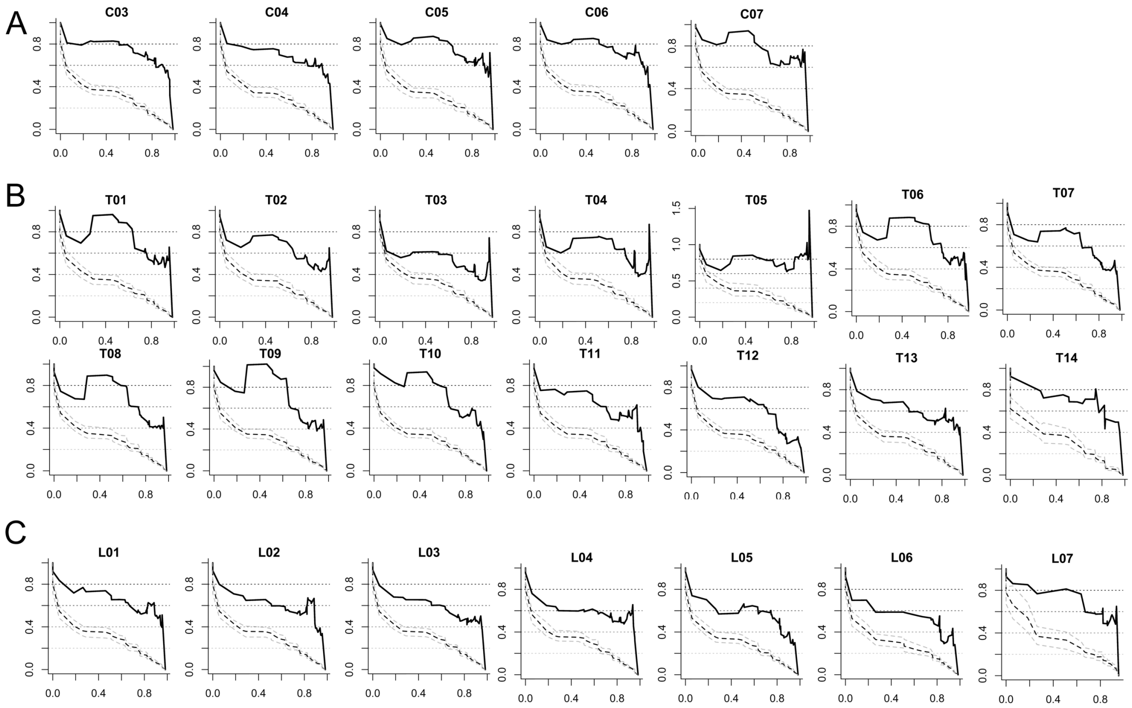

Supplementary Figure 2. Ecomorphological analysis of vertebral shape grouping vertebrae by number. A, including aquatic taxa; B, excluding aquatic taxa. In both cases the significance of R^2 (red-colour squares) for each vertebra is indicated by frame thickness; thick frames p-values < 0.01; thin frames p-values < 0.05; absence of frame indicates p-values > 0.05 (grey squares).

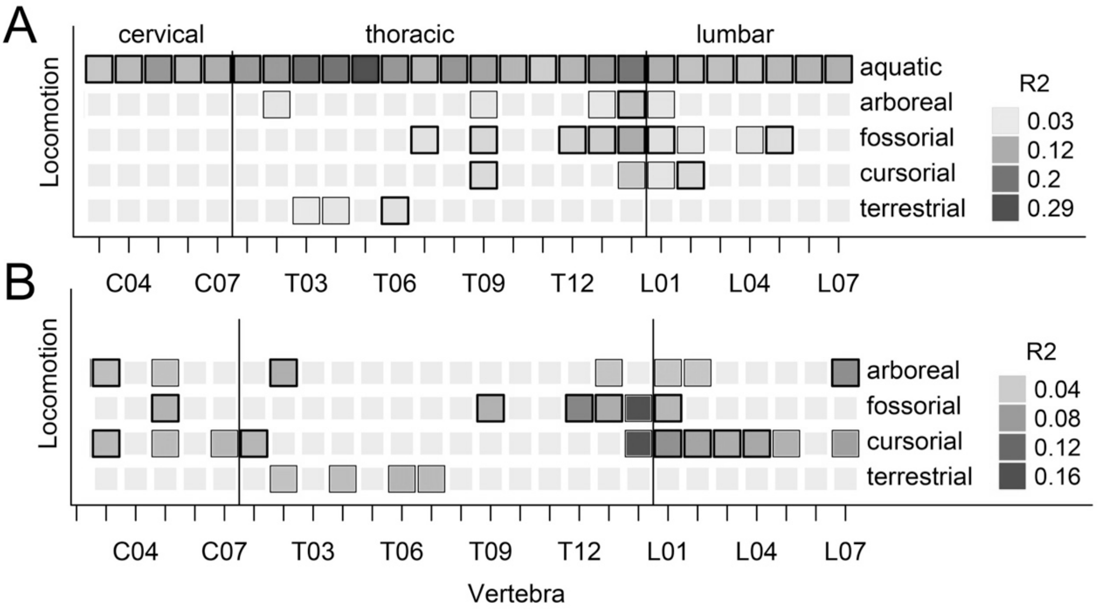

Supplementary Figure 3. Ecomorphological analysis of vertebral shape selecting vertebrae by position. A, including aquatic taxa; B, excluding aquatic taxa. In both cases the significance of R^2 (red-colour squares) for each vertebra is indicated by frame thickness; thick frames p-values < 0.01; thin frames p-values < 0.05; absence of frame indicates p-values > 0.05 (grey squares).

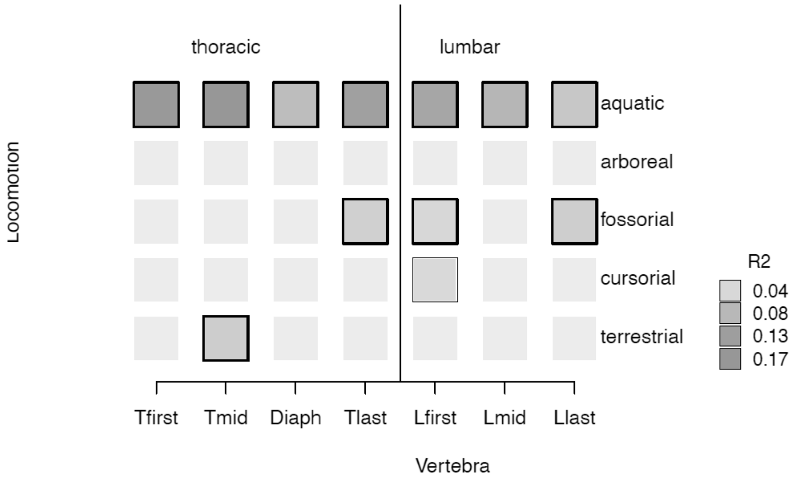

Supplement: Supplementary file 1 — Supplementary Information [file 42003_2021_2346_MOESM1_ESM.pdf]
