# Supplementary material for: Serial disparity in the carnivoran backbone unveils a complex adaptive role in metameric evolution
Source: Commun Biol. 2021 Jul 15;4:863. doi: 10.1038/s42003-021-02346-0 (PMC8282787; doi:10.1038/s42003-021-02346-0)
Supplement: Supplementary file 2 — Description of Additional Supplementary Files [file 42003_2021_2346_MOESM2_ESM.pdf]

Description for Additional Supplementary Files

File name: Supplementary Data 1

Description: Sampled species (specimen number; museum) and the ecological coding used to test the association between vertebral shape and the ecology of the species (locomotion) taken from the literature (see text for details). Abbreviations: UVA, Anatomical Museum of the Valladolid University (Spain); MNCN, National Museum of Natural Sciences (Madrid); NMS, National Museum of Scotland.

File name: Supplementary Data 2

Description: Results obtained from the Procrustes ANOVA to test the association between the ecology of taxa and the vertebral shape.

File name: Supplementary Data 3

Description: Results obtained from the Procrustes ANOVA to test the association between the ecology of taxa and vertebral shape excluding aquatic taxa.

File name: Supplementary Data 4

Description: Raw coordinates of the landmarks digitized in each vertebra that have been subject to analysis and constitute the source data underlying the graphs and charts presented in the main figures.
