# Supplementary material for: Serial disparity in the carnivoran backbone unveils a complex adaptive role in metameric evolution
Source: Commun Biol. 2021 Jul 15;4:863. doi: 10.1038/s42003-021-02346-0 (PMC8282787; doi:10.1038/s42003-021-02346-0)
Supplement: Supplementary file 7 — Reporting Summary [file 42003_2021_2346_MOESM7_ESM.pdf]

nature research

Corresponding author(s): Borja Figueirido

Last updated by author(s): Apr 16, 2020

Reporting Summary

Nature Research wishes to improve the reproducibility of the work that we publish. This form provides structure for consistency and transparency in reporting. For further information on Nature Research policies, see our Editorial Policies and the Editorial Policy Checklist.

Statistics

For all statistical analyses, confirm that the following items are present in the figure legend, table legend, main text, or Methods section.

n/a	Confirmed	
☐	☒	The exact sample size (n) for each experimental group/condition, given as a discrete number and unit of measurement
☐	☒	A statement on whether measurements were taken from distinct samples or whether the same sample was measured repeatedly
☐	☒	The statistical test(s) used AND whether they are one- or two-sided<br>*Only common tests should be described solely by name; describe more complex techniques in the Methods section.*
☐	☒	A description of all covariates tested
☒	☐	A description of any assumptions or corrections, such as tests of normality and adjustment for multiple comparisons
☐	☒	A full description of the statistical parameters including central tendency (e.g. means) or other basic estimates (e.g. regression coefficient) AND variation (e.g. standard deviation) or associated estimates of uncertainty (e.g. confidence intervals)
☐	☒	For null hypothesis testing, the test statistic (e.g. F, t, r) with confidence intervals, effect sizes, degrees of freedom and P value noted<br>*Give P values as exact values whenever suitable.*
☒	☐	For Bayesian analysis, information on the choice of priors and Markov chain Monte Carlo settings
☐	☒	For hierarchical and complex designs, identification of the appropriate level for tests and full reporting of outcomes
☐	☒	Estimates of effect sizes (e.g. Cohen's d, Pearson's r), indicating how they were calculated

Our web collection on statistics for biologists contains articles on many of the points above.

Software and code

Policy information about availability of computer code

Data collection	The landmarks in the 3D models were collected with the software landmark from IDAV (Wiley et al. 2005). Specified in REF. 47. Wiley, D. F., Amenta, N., Alcantara, D. A., Ghosh, D., Kil, Y. J., Delson, E., & Hamann, B. (2005). Evolutionary morphing (pp. 431-438). IEEE.
Data analysis	-Geomorph package of R. version 3.1.0. Citer in REF. 48: Adams, D. C., M. L. Collyer, and A. Kaliontzopoulou. (2019). Geomorph: Software for geometric morphometric analyses. R package version 3.1.0. https://cran.r-project.org/package=geomorph.<br>-3D models were edited with Meshlab. Specified in REF. 46: Cignoni, P., Callieri, M., Corsini, M., Dellepiane, M., Ganovelli, F., & Ranzuglia, G. (2008, July). Meshlab: an open-source mesh processing tool. In Eurographics Italian chapter conference (Vol. 2008, pp. 129-136).<br>-NLME package of R. version 3.1-139. Cited in REF. 65: 65. Pinheiro J, Bates D, DebRoy S, Sarkar D, R Core Team (2019). Nlme: Linear and Nonlinear Mixed Effects Models_. R package version 3.1-139, <URL: https://CRAN.R-project.org/package=nlme.<br>-CT scans were segmented in AVIZO : FEI Visualization Sciences Group. 2015. Avizo Lite 9.0.1. FEI, Hillsboro, OR.

For manuscripts utilizing custom algorithms or software that are central to the research but not yet described in published literature, software must be made available to editors and reviewers. We strongly encourage code deposition in a community repository (e.g. GitHub). See the Nature Research guidelines for submitting code & software for further information.

Data

Policy information about availability of data

All manuscripts must include a data availability statement. This statement should provide the following information, where applicable:

- Accession codes, unique identifiers, or web links for publicly available datasets
- A list of figures that have associated raw data
- A description of any restrictions on data availability

The data is uploaded as Supplementary Data in .zip file.

Field-specific reporting

Please select the one below that is the best fit for your research. If you are not sure, read the appropriate sections before making your selection.

- [ ] Life sciences
- [ ] Behavioural & social sciences
- [x] Ecological, evolutionary & environmental sciences

For a reference copy of the document with all sections, see nature.com/documents/nr-reporting-summary-flat.pdf

Ecological, evolutionary & environmental sciences study design

All studies must disclose on these points even when the disclosure is negative.

Study description

The paper investigates the relationship between morphological variability, evolutionary constraint and ecomorphological adaptation through the evolution of a metameric structure with functionally differentiated morphological regions in the vertebral column of mammalian carnivores. This multicomponent skeletal system has been extensively studied across all vertebrates because the formative roles of somitogenesis, somatic growth, and Hox gene expression are coupled with easily observable adult phenotypes. Despite this, there is the idea that the vertebral column is highly constrained to evolve by natural selection towards specific locomotory demands. However, this asseveration has been inferred from sparse anatomical samples, at least in mammals, providing little insight into local-scale (i.e. vertebra-to-vertebra) variation and its macroevolutionary importance.

Research sample

Here, we analyse 1097 three-dimensional models of vertebrae belonging to 44 species of mammalian carnivores: Acinonyx jubatus (NN;UVA), Ailuropoda melanoleuca (Z.1986.19; NHME), Ailurus fulgens (1802;UVA), Aonyx cinerea (2908;UVA), Arctictis binturong (5474;UVA), Atilax paludinosus (3253;UVA), Canis lupus (4589;UVA), Caracal caracal (1556;UVA), Chrysocyon brachyurus (Z.2015.177;NHME), Crocuta crocuta (4215;UVA), Cryptoprocta ferox (EA36.08; NHME), Cuon alpinus (7106; UVA), Cynictis penicillata (6730;UVA), Felis concolor (409;UVA), Felis silvestris (NN;UVA), Genetta genetta (1488;UVA), Genetta tigrina (3824;UVA), Herpestes ichneumon (7344;UVA), Hyena hyena (2981;UVA), Leptailurus serval (6070;UVA), Lutra lutra (2981;UVA), Lycaon pictus (1167;UVA), Lynx canadiensis (Z.2001.117.9;UVA), Martes foina (7342;UVA), Meles meles (NN;UVA), Mustela putorius (7203;UVA), Mustela vison (147;UVA), Nasua narica (5542;UVA), Neofelis nebulosa (Z.2001.8;NHME), Otaria byronia (Z.2004.179.1;NHME), Panthera leo (NN;UVA), Panthera pardus (1600;UVA), Panthera tigris (21574;MNCN), Paradoxurus hermaphroditus (4610;UVA), Phoca vitulina (M/180/02;NHME), Potos flavus (124;UVA), Procyon lotor (4264;UVA), Proteles cristatus (PH37.98;NHME), Speothos venaticus (Z.2015.96.4;NHME), Suricatta suricatta (3098;UVA), Tremarctos ornatus (Z.2008.34;NHME), Ursus arctos (Z.2003.41.1;NHME), Ursus maritimus (21570;MNCN), Vulpes vulpes (150;UVA). All the material is referred in Table S1.

Sampling strategy

No statistical methods were developed to predetermine sample size. We selected at least one column per species belonging to as much families as possible of the order Carnivora, covering a range of locomotor ecologies and body size, representatives of the entire spectrum.

Data collection

The vertebrae were scanned in 3D with either micro-computed tomography (CT) scanning or a NextEngine surface scanner. To capture the morphology of the vertebrae, we digitized 34 homologous landmarks on the cervical vertebrae (C03-C07), 32 homologous landmarks on the thoracic vertebrae (T01-T14), and 36 homologous landmarks on the lumbar vertebrae (L1-L7) (see Fig. 7; Appendix 1; Table S2). The landmarks were digitized with the software Landmark from IDAV (REF. 47) and the x,y,z coordinates of each landmark were exported as a Text file. The data was collected by the coauthor David Velasco.

Timing and spatial scale

The data was collected From January to August 2016.

Data exclusions

No data were excluded from the analysis.

Reproducibility

We do not have experiments in our paper.

Randomization

Organisms were allocated into the ecological locomotory groups (terrestrial, cursorial, arboreal, aquatic, fossorial) following the literature. This is specified in Table S1 (Appendix 1). This classification was relevant to investigate the association of vertebral shape with the ecology of species.

Blinding

Blinding was not relevant in our study because we do not perform experimental procedures for data collection or analyses.

Did the study involve field work? [ ] Yes [x] No

Reporting for specific materials, systems and methods

We require information from authors about some types of materials, experimental systems and methods used in many studies. Here, indicate whether each material, system or method listed is relevant to your study. If you are not sure if a list item applies to your research, read the appropriate section before selecting a response.

Materials & experimental systems

n/a	Involved in the study
x	Antibodies
x	Eukaryotic cell lines
	x Palaeontology and archaeology
x	Animals and other organisms
x	Human research participants
x	Clinical data
x	Dual use research of concern

Methods

n/a	Involved in the study
x	ChIP-seq
x	Flow cytometry
x	MRI-based neuroimaging

Palaeontology and Archaeology

Specimen provenance

UVA, Anatomical Museum of the Valladolid University (Spain); Dr. Francisco J. Pastor.
MNCN, National Museum of Natural Sciences (Madrid); Dr. Markus Bastir.
NHME, Natural History Museum of Edimburgh. Dr. Zena Timons.
All these curator have allowed us to use the data for academic purposes (published papers and teaching stuff). All of them between January-August 2016.

Specimen deposition

All the specimens are deposited in the collections aforomentioned above.

Dating methods

No new dates are provided

☐ Tick this box to confirm that the raw and calibrated dates are available in the paper or in Supplementary Information.

Ethics oversight

No ethical was required as there is not dating methods.

Note that full information on the approval of the study protocol must also be provided in the manuscript.
